# Evaluation of different solvents to extract antibacterial compounds from jalapeño peppers

**DOI:** 10.1002/fsn3.423

**Published:** 2016-08-31

**Authors:** Karleigh Bacon, Renee Boyer, Cynthia Denbow, Sean O'Keefe, Andrew Neilson, Robert Williams

**Affiliations:** ^1^ Department of Food Science and TechnologyVirginia Polytechnic Institute and State UniversityBlacksburgVAUSA; ^2^ Department of Plant Pathology, Physiology and Weed ScienceVirginia Polytechnic Institute and State UniversityBlacksburgVAUSA

**Keywords:** Antibacterial, *Capsicum annuum*, extraction, food‐borne pathogens, jalapeño, solvent

## Abstract

Chili peppers (*Capsicum* spp.) may possess antibacterial properties and have potential to be used in foods as antimicrobial. The complete chili pepper extract should be evaluated to determine which compounds are responsible for the antimicrobial activity. Extraction of compounds from the pepper is completed using a solvent. The type of solvent used for extraction influences which compounds are isolated, therefore the best extraction method needs to be determined. The purpose of this study was to identify which solvent is most successful at extracting unknown antibacterial compounds from jalapeño peppers. Fresh jalapeño peppers were chopped, weighed, and blended with a solvent (sterilized hot water, 70% methanol, 95% methanol, 70% ethanol, or 95% ethanol) at a 1:1 ratio (g/g) until the mixture was homogenized, followed by shaking for 15 min. The slurry was centrifuged; supernatant was removed and used for antibacterial testing against *Listeria monocytogenes*,* Escherichia coli* O157:H7, and *Salmonella enterica*. The diameter of growth inhibition was measured and statistically evaluated using ANOVA to determine the extract with the greatest antimicrobial activity. Solvents were tested alone as a control. There was greater bacterial inhibition from extracts created with methanol and ethanol than hot water. *Listeria monocytogenes* was significantly more susceptible to the extracts than *E. coli* or *Salmonella* isolates. Each solvent extract was then analyzed using high‐performance liquid chromatography (HPLC) and fractions (A–G) were collected and used for subsequent disk diffusion analysis against *L. monocytogenes*. Fractions E and F (eluded between 20 and 30 min) exhibited the most antibacterial activity. There were no differences between solvents used (*p* = .05). Further investigation into specific compounds within these extracts will be completed in the future.

## 1 Introduction

The food industry often relies on food additives to suppress microbial growth (Davidson & Taylor, 2007). Recently, there has been a resurgence of interest in natural antimicrobial compounds due to consumer concern for all‐natural or organic food products. Foods are commonly preserved by compounds such as nitrite, sodium benzoate, and sodium metabisulfite that have been tested and proven safe (Gould & Russell, 2003). However, there are occasional reports of allergic reactions to these preservatives, and even potential formation of carcinogenic byproducts like nitrosamines from nitrite (Roller & Seedhar, 2002). Essential oils isolated from some plant sources have been found to be effective antimicrobial agents (Cerrutti, Alzamora, & Vidales, 1997; Nascimento, Locatelli, Freitas, & Silva, 2000; Ngarmasak et al., 2006; Rupasinghe, Boulter‐Bitzer, Ahn, & Odumeru, 2006), and there is ongoing research to identify more antimicrobial plant sources.

A small number of studies have reported antimicrobial activity from *Capsicum* species fruit (Cichewicz & Thorpe, 1996; Dorantes et al., 2000). Cichewicz and Thorpe (1996) report inhibitory effects of a number of *Capsicum* species fruit extracts against *Bacillus cereus, B. subtilis, Clostridium sporogenes, Cl. tetani*, and *Streptococcus pyogenes* using a disk diffusion assay (Cichewicz & Thorpe, 1996). Jalapeño fruit extract, specifically, has been reported to be inhibitory to *S. pyogenes, Cl. sporogenes*, and *Cl. Tetani*; (Cichewicz & Thorpe, 1996). However, when these results were compared with trials using a commercially produced capsaicin (60 and 98% purity), no antimicrobial activity was found (Cichewicz & Thorpe, 1996). This suggests that antimicrobial activity of the extract is likely caused by other compounds, or another compound that acts synergistically with capsaicin. However, Molina‐Torres, Garcia‐Chavez, and Ramirez‐Chavez (1999) demonstrated that commercial capsaicin was strongly inhibitory against growth of *B. subtilis* and slowed the growth of *Escherichia coli* and *Pseudomonas solanacearum* slightly at some concentrations.

In another study, extracts of *C. annuum* varieties (habanero, serrano, pimiento morrón) inhibited growth of *Listeria monocytogenes*,* Staphylococcus aureus*,* Salmonella enterica* Typhimurium, and *B. cereus* (Dorantes et al., 2000). *L. monocytogenes* was the most sensitive to the extracts, followed by *B. cereus, S. aureus*, and *S. enterica* Typhimurium. The extracts were separated using reverse‐phase high‐performance liquid chromatography (HPLC) analysis to determine the content of compounds found in the capsaicinoid pathway for each pepper type. The content of phenylalanine, caffeic acid, coumaric acid, ferulic acid, cinnamic acid, capsaicin, and dihydrocapsaicin was all determined, and tested as inhibitors for growth of the four bacteria. Capsaicin and dihydrocapsaicin did not show inhibitory effects on the bacteria, but coumaric and cinnamic acids did show inhibitory affect (Dorantes et al., 2000). It is suggested that cinnamic acid may be a primary cause antimicrobial effects by inhibiting glucose uptake and ATP production within a bacterial cell (Dorantes et al., 2000).

These studies used different methods to extract the compounds from the fruit prior to evaluating antimicrobial activity of the extract. Methods include using distilled water with heat, distilled water with no heat, and various solvents. Using different methods can make the results from different studies difficult to compare. The purpose of this study was to determine the effect of different solvents (and their concentration) for creating jalapeno pepper extracts intended to be used in antimicrobial assays. The extraction method with the best results will be used in future antimicrobial assays to identify compounds from extracts with the greatest antimicrobial activity.

## 2 Materials and Methods

### 2.1 Bacterial cultures and culture conditions

Fifteen bacterial cultures were used in this study; five *Listeria monocytogenes*, five *Escherichia coli* O157:H7, and five *Salmonella enterica* isolates. Details of each strain used can be found in Table 1. Bacterial cultures were preserved in Tryptic Soy broth (TSB, Bacto, Difco, Becton Dickinson, Sparks, MD) containing 30% glycerol and stored at −80°C until use. Cells were activated by three successive 24 hr transfers into TSB and incubated at 37°C. Activated cells were centrifuged (Sorvall Legend RT+, Thermo Scientific, Waltham, MA) at 2000*g* for 10 min at 22°C, the pellet resuspended in 0.1% buffered peptone water, and washed twice more to yield a bacterial cocktail of approximately 8.0 log CFU/ml. Cultures were diluted 10‐fold into sterile peptone water to yield a concentration of approximately 7.0 log CFU/ml. This dilution was used in the disk diffusion assays.

**Table 1:** Bacterial strains and identification methods used in this study

Genus	Species/serovar	Source	Culture identification methods
*Salmonella*	*enteric* Saintpaul	UGA^a^‐jalapeño outbreak	XLT‐4 agar (Difco, Sparks, MD)API 20E (bioMérieux, Marcy E'toile, France)*Salmonella* Latex Agglutination
	*enteric* Anatum K2669	CDC^b^‐tomato	
	*enteric* Baildon	UGA^a^‐lettuce/tomato	
	*enteric* Newport 1893	CDC^b^‐tomato	
	*enteric* Javiana 2675	CDC^b^‐tomato	
*Escherichia*	*coli* O157:H7 H1730	UGA^a^‐lettuce	Sorbitol MacConkey agar (Difco, Sparks, MD)API 20ERIM *E. coli* Latex Agglutination
	*coli* O157:H7 F4546	UGA^a^‐alfalfa sprouts	
	*coli* O157:H7	UGA^a^‐cider	
	*coli* O157:H7 994	UGA^a^‐ beef	
	*coli* O157:H7 E0019	UGA^a^‐beef	
*Listeria*	*monocytogenes* ScottA	UGA^c^	Oxford medium base with Modified Oxford antimicrobic supplement (Difco, Sparks, MD)API *Listeria* (bioMérieux, Marcy E'toile, France)*Listeria* Latex Agglutination
	*monocytogenes* V7	CDC^b^	
	*monocytogenes* L‐CDC	CDC^b^	
	*monocytogenes* D43	Unknown	
	*monocytogenes* 2289	Unknown	

a Provided by Dr. L. R. Beuchat at the University of Georgia, Griffin, GA;

b Provided by the Center for Disease Control and Prevention, Atlanta, GA;

c Provided by Dr. R E. Brackett while at the University of Georgia, Griffin, GA.

### 2.2 Preparation of jalapeño extracts using no solvent

Extract made using no solvent was prepared following procedures outlined by Cichewicz and Thorpe (1996) with modifications. Fresh jalapeño peppers were purchased from a local grocery store in Blacksburg, VA, and rinsed with 100 ppm chlorine water for 2 min while shaking by hand. Peppers were then rinsed with sterile water (22°C) and diced with a sterile knife. Ten jalapeño peppers (approximately 200 g) were placed into a Waring blender (Waring, New Hartford, Conn.) and blended until a homogenous slurry was obtained (approx. 1 min). The slurry was placed into a filter lined stomacher bag and 50 ml of filtered liquid extract was removed from the bag. The extract was placed into a centrifuge tube and centrifuged at 15,000*g* for 10 min. The supernatant was collected and centrifuged twice more under the same conditions. The supernatant was removed and passed through a 0.45 μm pore size filter to sterilize (Whatman Inc., Piscataway, NJ). Extracts were used immediately for disk diffusion assays following preparation.

### 2.3 Preparation of jalapeño extracts using a solvent

Solvents used for this study were boiling (98°C) water, aqueous ethanol (70% and 95% (v/v)), and aqueous methanol (70% and 95% (v/v)). Jalapeños were purchased, rinsed, and diced as described above. Jalapeños were added to a Waring blender, and solvent was added at a ratio of 1:1 (wt/wt). Jalapeños and solvent were blended for approximately 1 min until a homogenous slurry was obtained. The slurry was poured into a 500 ml beaker. Controls for each solvent extract were prepared as described above, replacing jalapeño weight with charcoal and omitting the blending step. Both experimental and control beakers were covered with aluminum foil and placed in an orbital shaker for 24 hr at room temperature. After 24 hr, extracts were poured into filter‐lined stomacher bags and prepared as described above. Extracts were immediately used for disk diffusion assays following preparation.

### 2.4 Reverse‐phase high‐performance liquid chromatography of jalapeño extracts

Analyses of the solvent and nonsolvent extracts were performed using a reverse‐phase HPLC technique employing an Agilent 1200 Series HPLC (Santa Clara, CA) consisting of degasser, quaternary solvent pump, autosampler with refrigeration, column oven, and a diode array detector and a Phenomenex (Torrance, CA) Luna 5μ C18 (250 × 4.6 mm) column with a Phenomenex Security Guard column. A gradient consisting of two solvents, solvent A (0.1% acetic acid in water) and solvent B (0.1% acetic acid in acetonitrile), was used. Flow rate was 1.0 ml/min. The sample injection volume was 100 μl. UV absorbance was recorded at 254 and 280 nm. Compounds from the jalapeño extract were collected as they eluted from the HPLC column in 5 min increments (0–5 min: Fraction A; 5–10 min: Fraction B, etc.) into clean glass centrifuge tubes. This was repeated for each of the different solvent extracts.

### 2.5 Preparation of HPLC fractions for disk diffusion assays

Collected fractions were placed under a fume hood and the mobile phase was evaporated from fractions using a gentle stream of nitrogen gas. One ml of sterile deionized water was then added to each tube (to dilute any residual mobile phase), tubes were capped, and placed into the freezer (−18 ± 2°C) for approximately 3 hr until samples were frozen solid. Caps were then removed from tubes and cheese cloth was secured over the tube openings with a rubber band. Samples were placed into a freeze dryer (Virtis, The Virtis Company Inc., Gardiner, New York) and dried for approximately 18 hr until all liquid was removed from the samples and an off‐white powder could be detected at the bottom of the tubes. Fractions were resuspended in sterile deionized water to achieve a concentration of 100 ppm. Fractions were used immediately, or stored at 4°C until ready for use in antimicrobial disk diffusion assays.

### 2.6 Disk diffusion assays

A disk diffusion assay was performed following the method of Vigil, Palou, Parish, and Davidson (2005) with some modifications. Whatman #2 filter paper (Whatman Inc., Piscataway, NJ) was used in this assay. A hole punch was used to produce 6.5‐mm‐diameter filter disks. The disks were collected and autoclaved prior to use. Bacterial cultures (previously described; 7.0 log cfu/ml diluted in 0.1% sterile peptone water) were spread plated (0.1 ml) onto Tryptic Soy Agar (TSA, Bacto, Difco, Becton Dickinson, Sparks, MD). Plates were allowed to dry for 10 min. Flame‐sterilized tweezers were used to place filter disks onto inoculated TSA plates, one disk in each of four equal quadrants. Each disk on the TSA was then impregnated with 10 μl of either liquid extract treatment or control (two control disks and two extract disks per plate). For controls, 100 μl of relevant solvent was injected into the HPLC, collected, and fractions collected and processed as described above. Two plates were prepared for each unique culture and solvent combination (*n* = 4). Plates were inverted and incubated for 24 hr at 37°C. Zones of inhibition were measured in mm with a digital caliper. Each experiment was run three times (*N* = 12).

In addition, a filter disk assay was performed in order to rule out the antimicrobial activity attributed to the pH of the extract. The pH of the crude jalapeño extract was determined to be 5.67. Based on this measurement, an experimental sample was prepared by filter SDW with an adjusted pH of 5.67. A control test sample was made using SDW (pH 7.40). Both experimental and control samples were used for a disk diffusion assay, and inhibition results were compared.

### 2.7 Statistical analysis

The diameter of growth inhibition was statistically evaluated using one‐way ANOVA as well as Tukey's Honestly Significant Difference post hoc test to compare mean zones of inhibition for jalapeño extracts and controls. All analyses were performed using JMP 7.0 statistical software (SAS Institute, Cary, NC). Significance was defined as *p* < .05.

## 3 Results and Discussion

There was no inhibition observed due to pH of the jalapeño pepper slurry (data not shown). The jalapeño extracts evaluated all displayed antibacterial activity against one or more bacteria, with the exception of the extracts from the hot water extraction method which showed no inhibition. It is important to note, however, that the extract produced without solvent was more concentrated than the extracts produced with solvent due to the dilution effect of the solvent. This may explain why the extract obtained without using a solvent exhibited enhanced antibacterial activity compared to the hot water extract.

**Figure 1:**
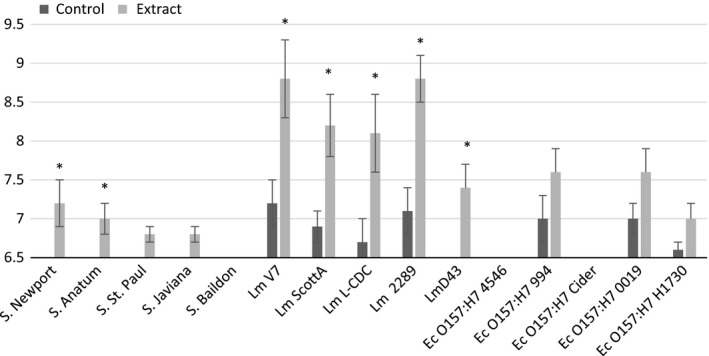
Mean zones of inhibition for bacterial cultures in association with jalapeño extract made with no solvent. Sterile deionized water was used for the control in both assays. Asterisks represent measurements where zones of inhibition for solvent extracts are significantly different than zones of inhibition for their respective controls. If no inhibition was seen, a value of 6.5 was assigned, which was the diameter of the disk used for the disk diffusion experiments

**Figure 2:**
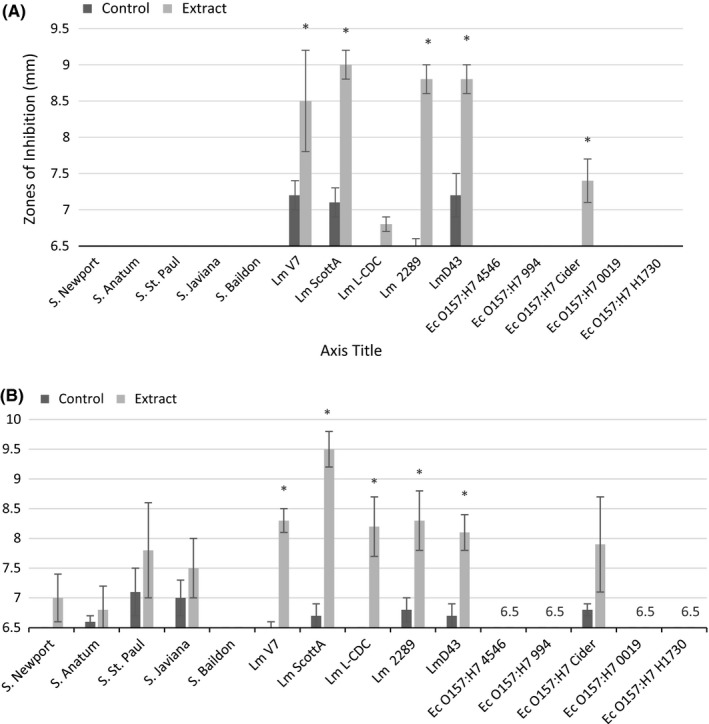
Mean zones of inhibition for bacterial cultures in association with jalapeño extract made with (A) 70% methanol solvent and (B) 95% methanol solvent. Controls are the solvent with water replacing jalapeño extract. Asterisks represent measurements where zones of inhibition for solvent extracts are significantly different than zones of inhibition for their respective controls. If no inhibition was seen, a value of 6.5 was assigned, which was the diameter of the disk used for the disk diffusion experiments

**Figure 3:**
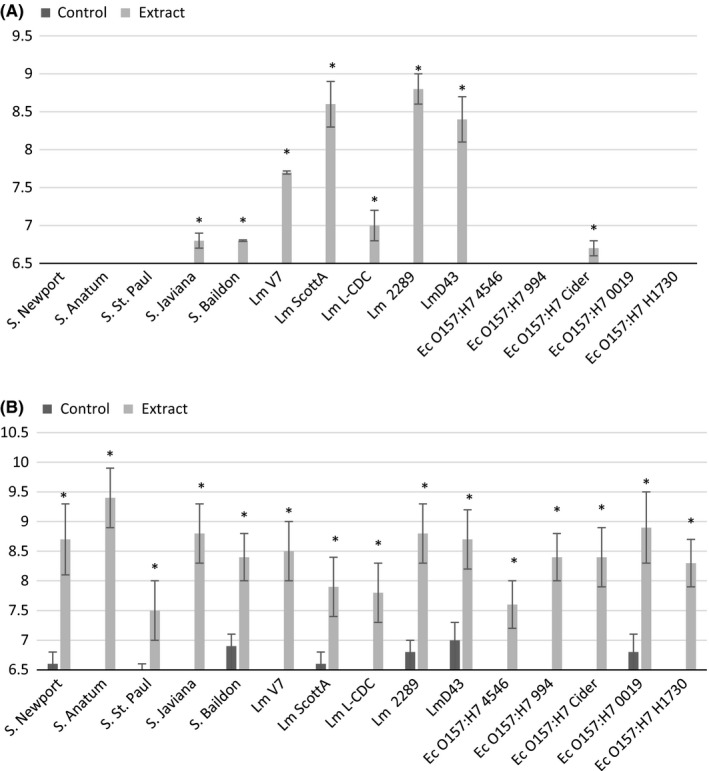
Mean zones of inhibition for bacterial cultures in association with jalapeño extract made with (A) 70% ethanol solvent and (B) 95% ethanol solvent. Controls are the solvent with water replacing jalapeño extract. Asterisks represent measurements where zones of inhibition for solvent extracts are significantly different than zones of inhibition for their respective controls. If no inhibition was seen, a value of 6.5 was assigned, which was the diameter of the disk used for the disk diffusion experiments

The mean diameters of the inhibition zones of all solvent extracts against *L. monocytogenes*,* S. enterica*, and *E. coli* are shown in Figures 1, 2, 3. Our results showed that *L. monocytogenes* cultures were the most consistently inhibited by the extracts, producing measurable zones of inhibition for each solvent tested except for hot water. The largest zones of inhibition associated with *L. monocytogenes* were observed with jalapeño extract obtained without solvent, as well as with 95% methanol and ethanol extracts. Dorantes et al. (2000) also found that *L. monocytogenes* was the most sensitive to extracts of different *C. annuum* peppers when compared to *B. cereus, S. aureus,* and *S. enterica* Typhimurium. Methanol was the most promising solvent for extracting anti‐Listerial compounds based on differences between experimental and control zones of inhibition. Both concentrations of the methanol solvent tested were successful, but the 95% methanol solvent resulted in the greatest differences in inhibition between controls and extracts (Fig. 2).

The extract produced with 95% ethanol significantly inhibited all of the bacterial cultures evaluated (Fig. 3B). Controls were run in parallel to ensure inhibition could be properly attributed to the extract rather than the alcohol solvent. Inhibition was seen with some of the controls for this experiment, but it was not significant (Fig. 3B). Although it is possible that the ethanol solvent is extracting antimicrobial compounds that are accounting for the large zones of inhibition observed, the use of 95% ethanol must be cautioned due to the antibacterial activity of the control. For this reason, 95% ethanol will not be used further to pursue studies in this area. Generally, all other extracts produced did not significantly reduce growth of *E. coli* O157:H7 or *S. enterica* isolates with the exception of *S*. Newport and Anatum, which were inhibited by extract produce without solvent (Fig. 1A); *S*. Javiana and Baildon, which were inhibited by extract produced with 70% ethanol (Fig. 3A); and *E. coli* O157:H7 Cider, which was inhibited by extracts produced with 70% methanol and 70% ethanol (Figs. 23A–A). The inhibition response to extracts was not uniform across members of the same bacterial genus. The most sensitive species for each genus were *L. monocytogenes* 2289, *Salmonella* Anatum, and *E. coli* Cider, respectively.

**Figure 4:**
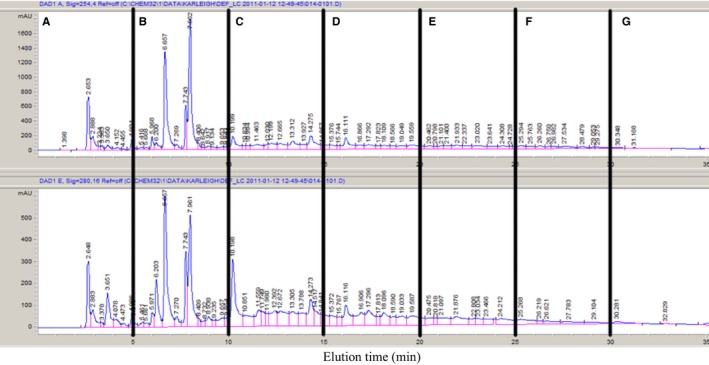
Reverse‐phase high‐performance liquid chromatography UV chromatograms (top: 254 nm, bottom: 280 nm) of jalapeño extract with vertical indicators of three fractions collected. Fractions were collected every 5 min and assigned alphabetical labels based on time of elution

**Figure 5:**
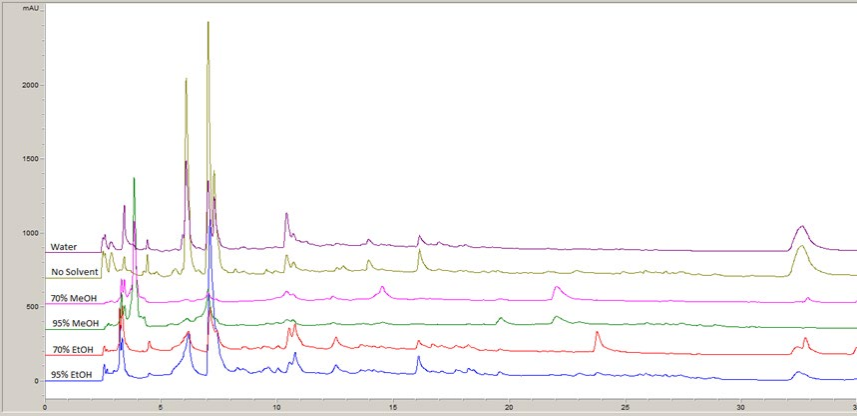
Representative high‐performance liquid chromatography chromatogram (280 nm) of jalapeño extracts using different solvents for extraction

The extracts were analyzed with HPLC to show the differences in compound extraction achieved by using different solvents. An example of one of the HPLC chromatograms produced can be seen in Figure 4. The extraction methods did cause some variation between chromatograms (Fig. 5). Again, it is necessary to note that the nonsolvent extract was more concentrated than the solvent extracts, which is clear in Figure 5. However, the overall chromatograms look similar in terms of qualitative profile. The main differences were quantitative in nature. In order to test whether the different solvents made a difference when the extracts were collected as fractions off the HPLC column, fractions of each solvent extract were collected and tested against *L. monocytogenes* using the disk diffusion assay. *L*. *monocytogenes* was chosen due to the sensitivity shown in disk diffusion assays with the unfractionated compounds.

**Figure 6:**
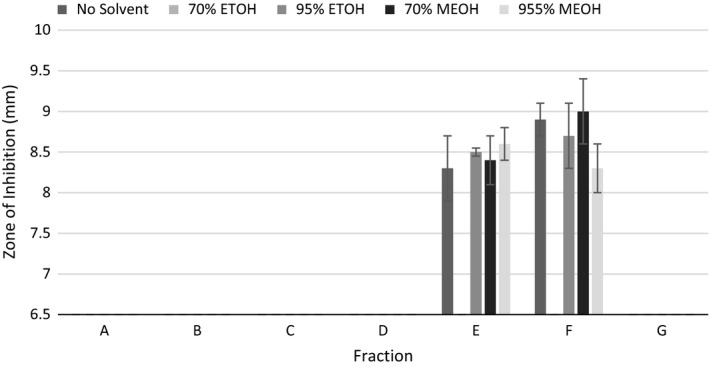
Comparison of the mean zones of inhibition for *Listeria monocytogenes* and fractionated jalapeño extracts using different solvents. Error bars represent standard error of the mean. If no inhibition was seen, a value of 6.5 was assigned, which was the diameter of the disk used for the disk diffusion experiments

Results for the disk diffusion assay using the fractionated extracts for *L. monocytogenes* revealed that Fraction E and Fraction F (corresponding to 20–25 and 25–30 min elution, respectively) contained the most active inhibiting compounds (Fig. 6). There was no inhibition observed for the other fractions (A–D, and G; Fig. 5). Fractions of E and F produced using the 70% MeOH solvent did not produce any zones of inhibition for *L. monocytogenes*. This was unexpected, as 70% MeOH Fraction E appeared to have a unique compound (eluting at ~24 min, Fig. 4). Extracts of Fractions E and F produced using all other solvents significantly decreased growth of *L. monocytogenes*, but there were no significant differences between extraction methods.

The fractionation process further confirmed that the active compounds are likely minor constituents, as very small or no peaks were observed in the 20–30 min elution window (or are compounds that absorb at other wavelengths, such as carotenoids) corresponding to Fractions E and F. Therefore, mass spectrometry analysis may prove useful for identifying these compounds.

Furthermore, fractionation confirmed the relative hydrophobic nature of the active compounds, as they eluted relatively late in the gradient. While these extracts were prepared with organic solvents, the solvents were relatively polar and contained large amounts of water. Because the same amount of inhibition was displayed for the extract using no solvent as the extracts that were made using solvents, it is recommended that future studies evaluating the antimicrobial activity of jalapeño extract be conducted without the use of a solvent to prepare the extract. However, the potential exists for jalapeños to contain more hydrophobic compounds with potent antimicrobial activity. These compounds may need to be extracted using very nonpolar solvents (in order of decreasing polarity: acetonitrile, ethyl acetate, acetone, methylene chloride, chloroform, chloroform, toluene, cyclohexane, and hexane). This may reveal another suite of extracts with distinct, and potentially greater, antimicrobial activities from those reported here.

Jalapeño peppers are a promising resource for natural antibacterial components, especially for inhibition of *L. monocytogenes*. Our study builds upon the research to indicate that jalapeño peppers contain potential antimicrobial compounds. Although a number of solvents were tested for extraction of these antimicrobial compounds, HPLC analysis and disk diffusion assays showed that there is little difference in the antimicrobial activities between the solvents used for extraction in this study. *Listeria monocytogenes* exhibited the highest sensitivity to the extracts in all the studies conducted. Therefore, further studies should focus on the isolation and identification of compounds that may be contributing to inhibition of pathogenic food‐borne bacteria, especially *L. monocytogenes*. Additionally, the creation of extracts with less polar solvents should be evaluated.

## Funding Information

No funding information provided.

## Conflict of Interest

The authors have no conflicts of interest to declare related to this work.
